# miR‐34b‐5p inhibition attenuates lung inflammation and apoptosis in an LPS‐induced acute lung injury mouse model by targeting progranulin

**DOI:** 10.1002/jcp.26274

**Published:** 2018-03-25

**Authors:** Wang Xie, Qingchun Lu, Kailing Wang, Jingjing Lu, Xia Gu, Dongyi Zhu, Fanglei Liu, Zhongliang Guo

**Affiliations:** ^1^ Department of Respiratory Medicine Shanghai East Hospital Tongji University School of Medicine Pudong Shanghai China

**Keywords:** acute lung injury, apoptosis, inflammation, miR‐34b‐5p, progranulin

## Abstract

Inflammation and apoptosis play important roles in the initiation and progression of acute lung injury (ALI). Our previous study has shown that progranulin (PGRN) exerts lung protective effects during LPS‐induced ALI. Here, we have investigated the potential roles of PGRN‐targeting microRNAs (miRNAs) in regulating inflammation and apoptosis in ALI and have highlighted the important role of PGRN. LPS‐induced lung injury and the protective roles of PGRN in ALI were first confirmed. The function of miR‐34b‐5p in ALI was determined by transfection of a miR‐34b‐5p mimic or inhibitor in intro and in vivo. The PGRN level gradually increased and subsequently significantly decreased, reaching its lowest value by 24 hr; PGRN was still elevated compared to the control. The change was accompanied by a release of inflammatory mediators and accumulation of inflammatory cells in the lungs. Using bioinformatics analysis and RT‐PCR, we demonstrated that, among 12 putative miRNAs, the kinetics of the miR‐34b‐5p levels were closely associated with PGRN expression in the lung homogenates. The gain‐ and loss‐of‐function analysis, dual‐luciferase reporter assays, and rescue experiments confirmed that PGRN was the functional target of miR‐34b‐5p. Intravenous injection of miR‐34b‐5p antagomir in vivo significantly inhibited miR‐34b‐5p up‐regulation, reduced inflammatory cytokine release, decreased alveolar epithelial cell apoptosis, attenuated lung inflammation, and improved survival by targeting PGRN during ALI. miR‐34b‐5p knockdown attenuates lung inflammation and apoptosis in an LPS‐induced ALI mouse model by targeting PGRN. This study shows that miR‐34b‐5p and PGRN may be potential targets for ALI treatments.

## Graphical abstract

miR‐34b‐5p participates in the development of acute lung injury by regulating progranulin.

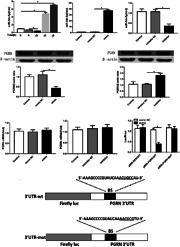

## 1 INTRODUCTION

Acute lung injury (ALI) and acute respiratory distress syndrome (ARDS) are severe clinical conditions that are seen in intensive care units worldwide, with high morbidity, and mortality in critical ill patients (Blank & Napolitano, 2011). Although significant advancements have been made regarding the therapeutic strategy and understanding of associated the respiratory physiology, the annual mortality for ARDS and ALI are still 40%, and the diseases lead to significant healthcare costs (Bocharov et al., 2016). ALI is a prevailing inflammatory lung disease characterized by exaggerated production of pro‐inflammatory mediators, infiltration of inflammatory cells, and apoptosis of alveolar epithelial cells (Cox et al., 2015; Liu, Zheng, Fan, Peng, & Su, 2016). Control of aberrant inflammation and apoptosis contributes substantially toward improving prognosis (Sureshbabu et al., 2016).

Progranulin (PGRN), which is expressed in epithelial cells and macrophages, is a secreted growth factor with seven cysteine‐rich repeats (He, Ong, Halper, & Bateman, 2003). Previous studies have shown that PGRN plays a critical role in a series of physiologic and pathological processes, including wound healing, inflammation, and apoptosis (Cerezo et al., 2015; Ma, Matsuwaki, Yamanouchi, & Nishihara, 2017; Zhou, Tang, et al., 2015). PGRN‐deficient mice display severe lung injury and fatal inflammatory responses when the mice are subjected to sepsis or endotoxic shock (Song et al., 2016; Zhou, Tang, et al., 2015). Consistently, we have found that PGNR expression is significantly down‐regulated in the bronchial alveolar lavage fluid (BALF) of mice with ALI and that administration of recombinant murine PGRN significantly reduces LPS‐induced lung inflammation (Guo et al., 2012). Although we have demonstrated that PGRN plays important roles in the development of ALI, the specific roles, and regulatory mechanisms of PGRN await further investigation.

MicroRNAs (miRNAs) are a class of naturally occurring small noncoding RNAs that comprise 19–22 nucleotides and that modulate both the stability and translation of targeted mRNA (Correia et al., 2017). MiR‐34b‐5p, which belongs to the miR‐34 family, is mainly expressed in the lung (Liang, Ridzon, Wong, & Chen, 2007). Aberrant expression of miR‐34b‐5p is linked to colorectal cancer and lung cancer (Jiang & Hermeking, 2017; Tanaka et al., 2012). Notably, recent studies have indicated that miR‐34b‐5p is prominently induced in inflammation‐related disorders, such as acute graft‐versus‐host disease, and intracranial aneurysm (Jalapothu et al., 2016; Li et al., 2012), suggesting the potential function of miR‐34b‐5p in inflammatory signaling pathway. However, neither the exact role of miR‐34b‐5p nor the regulatory mechanism have been defined in ALI.

In this study, we first explored the potential roles of PGRN in modulating ALI by using recombinant adenovirus containing the mouse PGRN gene or the mouse PGRN shRNA. Then, we performed bioinformatics analysis to screen potential miRNAs targeting PGRN in ALI and determined the expression levels of these miRNAs in the lung homogenates before and after ALI. We hypothesized that some miRNAs played protective roles while others played harmful roles and that these roles depended on miRNA‐mediated PGRN change. This study provides a novel strategy for the prevention and treatment of ALI.

## 2 MATERIALS AND METHODS

### 2.1 Animal model of ALI

Six‐to‐eight‐week adult male C57BL/6 mice were purchased from Shanghai Laboratory Animal Company and maintained in specific pathogen‐free conditions under controlled temperature and humidity. The mice were subjected to 1 mg/kg or 25 mg/kg of LPS (*Escherichia coli* O55:B5, Sigma, Saint Louis, MO) via intratracheal instillation to induce ALI and generate the ALI model (Park, Lee, Kim, & Yang, 2016). Animal experiment were approved by the Tongji University animal care and use committee.

### 2.2 Adenovirus gene delivery

Recombinant adenovirus containing the mouse PGRN gene (Ad‐PGRN) or the mouse PGRN shRNA(Ad‐PGRN‐shRNA) were purchased from Obio Company (Obio Technology, Shanghai, China). Adenovirus expressing no transgene was used as the negative control (Ad‐GFP). Seven days before ALI induction, a dose (1 × 10^9^ pfu) of adenovirus was intratracheally instilled into the mice (Wang, Wang, et al., 2016). The control mice were treated with either sterile saline or the control adenovirus (Ad‐GFP). The efficacy of inference was assessed with Western blot.

### 2.3 Lung histology and TUNEL staining

Mouse lungs from all groups were fixed with 4% paraformaldehyde, embedded, and cut into 4 μm sections. The sections were stained with hematoxylin and eosin (HE), and five microscopic fields were used to assess the lung injury score based on a previous study (Mrozek et al., 1997). TUNEL staining was performed with a commercial kit (Roche, Bern, Switzerland) based on the manufacturer's instructions.

### 2.4 Immunohistochemical staining

The expression of PGRN and the presence of neutrophils and macrophages in the lung sections were evaluated by immunohistochemical staining as described (Villar et al., 2015) with a PGRN antibody (Abcam, Cambridge, UK, Cat#ab191211), Gr‐1 (R&D Systems, Minneapplis, MN, Cat#MAB1037), and CD68 antibody (Abcam Cat#ab955).

### 2.5 Pulmonary edema level

The right upper lung tissues were weighed and then dried in an oven at 80°C. Forty‐eight hours later, the weight of each tissue was measured again to calculate wet‐to‐dry (W/D) ratio.

### 2.6 Bronchial alveolar lavage fluid analysis

Bronchial alveolar lavage (BAL) was conducted as previously described (Kral‐Pointner et al., 2017). Briefly, 1 ml of PBS was injected into the lungs via the trachea and then carefully drawn out three times. BAL cells were precipitated, resuspended, and used for a cell count. The supernatant was used to detect BAL proteins and inflammatory mediators.

### 2.7 Caspase‐3 and MPO activity assays

The caspase‐3 and myeloperoxidase (MPO) activities in the lung homogenates were measured with commercial kits (Beyotime and Nanjing Jiancheng, Tianjin and Nanjing, respectively, China) based on the manufacturer's protocols.

### 2.8 Cytokine measurements

The levels of tumor necrosis factor‐α (TNF‐α), interleukin‐6 (IL‐6), and interleukin‐1β (IL‐1β) in the BALF were measured with ELISA kits (eBioscience, San Diego, CA) according to the manufacturer's protocols.

### 2.9 MicroRNA‐34b‐5p antagomir transfection

The miR‐34b‐5p antagomir (5′‐ACAAUCAGCUAAUUACACUGCCU‐3′) and negative control antagomir were purchased from GenePharma (Shanghai, China). The Entranster in vivo transfection reagent was gained from Engreen Biosystem Co (Beijing, China). The transfection protocol was performed based on the manufacturer's instructions. Briefly, we first prepared a nucleic acid dilution by dissolving 50 µg of the miR‐34b‐5p antagomir or negative control antagomir in 50 µl of a sterilized double‐distilled H_2_O. Then, we added 50 µl of a sterile 10% glucose solution and mixed the solution well. Next, we prepared a transfection reagent dilution by dissolving 25 µl of the transfection reagent dilution of transfection reagent in 50 µl of the sterile 10% glucose solution and in 25 µl of the sterilized double‐distilled H_2_O, and the solution was mixed well. Finally, the nucleic acid dilution and transfection reagent dilution were mixed (1:1) to form working solution. For each mouse (20–25 g), we injected 200 µl of the working solution through the tail vein. The efficacy of the interference in vivo was evaluated by quantitative real‐time PCR (qRT‐PCR).

### 2.10 Luciferase reporter assay

The wild‐type (WT) 3′‐UTR of the PGRN cDNA was synthesized with PCR and cloned into the MluI and HindIII site of the pMIR‐REPORT Luciferase miRNA target expression vector (Obio Technology) to generate WT‐PGRN 3′‐UTR. This vector contained the Renilla and firefly luciferase genes. The mutant variant of the PGRN 3′‐UTR was generated based on the WT‐PGRN 3′‐UTR by mutating six nucleotides that could potentially bind to miR‐34b‐5p, and the resulting vector was named MUT‐PGRN 3′‐UTR. These vectors (the pMIR‐REPORT plasmid, WT‐PGRN 3′‐UTR, or MUT‐PGRN 3′‐UTR) and the miR‐34b‐5p mimic or miR‐negative control (NC) were transiently transfected into 293T cells using the Lipofectamine 3000 reagent (Invitrogen, Carlsbad, CA). Luciferase activity was measured with the Dual‐Luciferase Reporter Assay System (Promega, Madison, WI) after transfection for 48 hr.

### 2.11 Cell culture and transfection

RAW264.7 cells (passage no. 10) were purchased from the cell bank of the Chinese Academy of Science (Shanghai, China). RAW264.7 cells were cultured in Dulbecco's modified Eagle's medium (DMEM) supplemented with 10% fetal bovine serum and 1% penicillin/streptomycin and maintained at 37°C in a humidified incubator containing 5% CO_2_. Transfections were performed with Lipofectamine 3000 per the small‐interfering RNA (siRNA) or miRNA mimic and inhibitor transfection protocols. The transfection efficiency was measured by qRT‐PCR and Western blot. The miR‐34b‐5p mimic, control mimic, control inhibitor, miR‐34b‐5p inhibitor, and siRNAs against PGRN were synthesized by GenePharma. The sequences used were as follows: PGRN siRNA 5′‐GCUUCCAGAUGUCAGAUAATT‐3′ (sense), 5′‐UUAUCUGACAUCUGGAAGCTT‐3′ (antisense); miR‐34b‐5p mimic 5′‐AGGCAGUGUAAUUAGCUGAUUGU‐3′ (sense), 5′‐AAUCAGCUAAUUACACUGCCUUU‐3′ (antisense), and inhibitor (5′‐ACAAUCAGCUAAUUACACUGCCU‐3′).

### 2.12 RNA extraction and quantitative real‐time PCR

The miRNA and total RNA were extracted from cells or lungs using commercial kits (TIANGEN, Beijing, China) based on the manufacturer's protocols. miRNA and mRNA expression was measured using a SYBR Green Real‐Time PCR System (ABI, Tampa, FL). U6 and β‐actin were used as the internal controls. The primers are shown in Table 1. Relative gene expression was calculated and normalized using the 2^−ΔΔCt^ method.

**Table 1:** RT‐PCR primer sequences

Primer	Sequence
β‐actin	Forward: 5′‐TGTCCACCTTCCAGCAGATGT‐3′
	Reverse: 5′‐GCTCAGTAACAGTCCGCCTAGA‐3′
IL‐1β	Forward: 5′‐AGTTGACGGACCCCAAAAGAT‐3′
	Reverse: 5′‐GTTGATGTGCTGCTGCGAGA‐3′
TNF‐α	Forward: 5′‐CCCTCACACTCAGATCATCTTCT‐3′
	Reverse: 5′‐GCTACGACGTGGGCTACAG‐3′
IL‐6	Forward: 5′‐TAGTCCTTCCTACCCCAATTTCC‐3′
	Reverse: 5′‐TTGGTCCTTAGCCACTCCTTC‐3′
PGRN	Forward: 5′‐ATGTGGGTCCTGATGAGCTG‐3′
	Reverse: 5′‐GCTCGTTATTCTAGGCCATGTG‐3′
MiR‐34b‐5p	Forward: 5′‐CGAGGCAGTGTAATTAGCTGATTGT‐3′

### 2.13 Protein preparation and Western blot

Total protein from the lungs and cells was extracted with RIPA buffer (Beyotime, Tianjin, China). Thirty micrograms of protein was loaded onto SDS‐PAGE gels and then transferred to a PVDF membrane. Membranes were blocked with 5% milk in TBS‐T for 1 hr and incubated overnight (4°C) with antibodies against PGRN (Abcam, Cat#ab191211), cleave‐caspase‐3 (Cell Signaling Technology, Beverly,MA, Cat#9661S), TNF‐α (Cell Signaling Technology, Cat#11948), IL‐1β (Cell Signaling Technology, Cat#12242), IL‐6 (Cell Signaling Technology, Cat#12912), phospho‐NF‐κB p65 (Cell Signaling Technology, Cat#3033), NF‐κB p65 (Cell Signaling Technology, Cat#8242), and β‐actin (Proteintech, Chicago, IL, Cat#66009‐1‐Ig). Membranes were washed in TBS‐T and incubated with HRP‐conjugated anti‐rabbit or anti‐mouse secondary antibodies for 2 hr at room temperature. The protein band was visualized with an ECL system (BioRad, Hercules, CA).

### 2.14 Statistical analysis

Data were reported as the mean ± SD. The differences between multiple groups were examined with student's *t*‐test following one‐way ANOVA. *p *< 0.05 was considered to be statistically significant.

## 3 RESULTS

### 3.1 PGRN expression in the lungs of an LPS induced ALI

**Figure 1:**
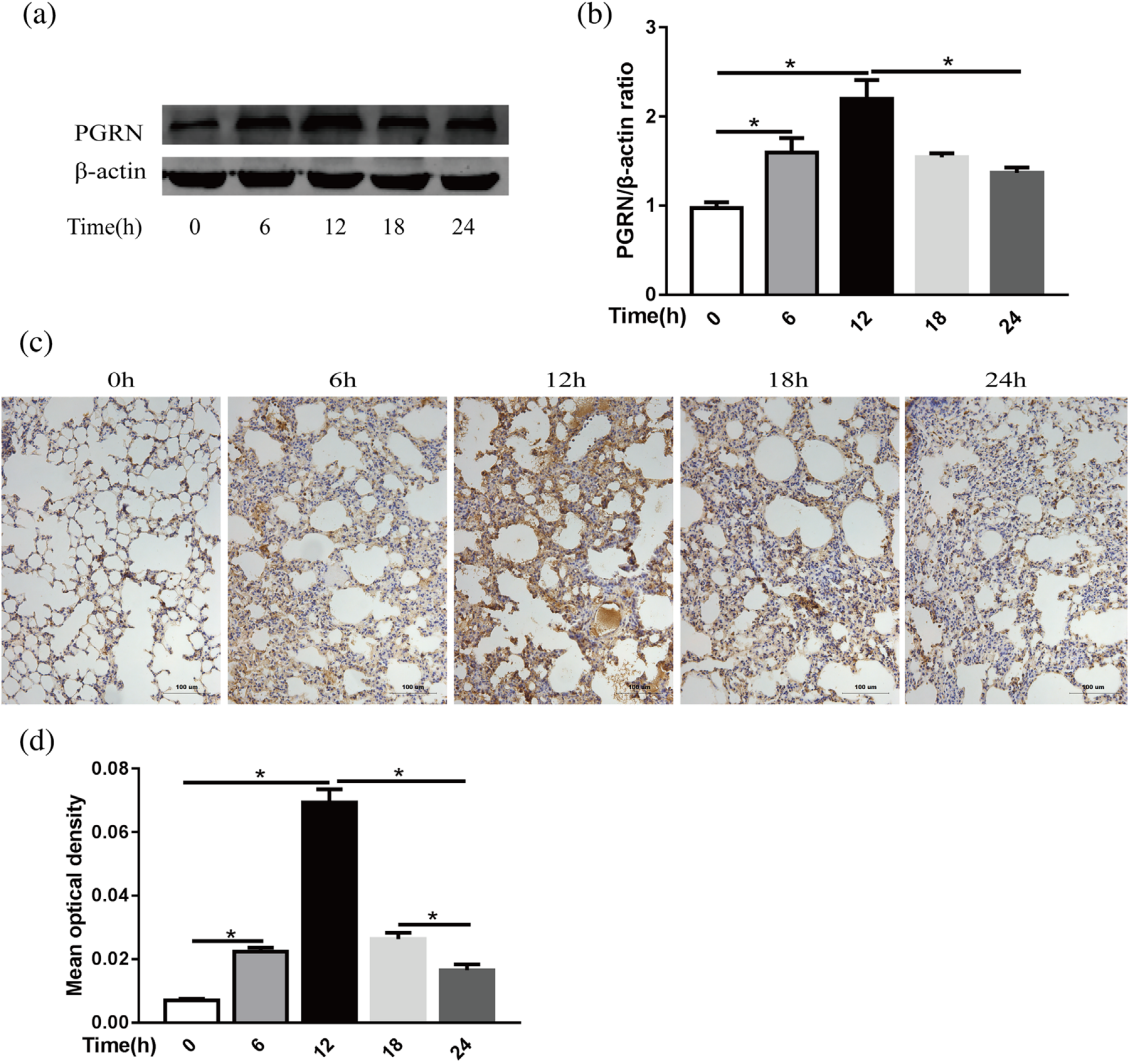
PGRN expression in whole lungs at different times after LPS injection in mice. (a) PGRN protein levels in mouse lungs at different times after administration of LPS (1 mg/kg) were evaluated by Western blot, and β‐actin was used as a loading control. (b) Quantitation of the PGRN/β‐actin ratio in the lungs from mice with LPS injections at different times. (c) Representative images of PGRN expression in lung sections after administration of LPS per immunohistochemical staining. Scale bar = 100 μm, *n* = 4 (d) Quantitation of the mean optical density of PGRN in the lung from mice with LPS injection at different times. *p < 0.05. Data are presented as the mean ± SD

To assess endogenous PGRN protein expression during ALI, LPS intratracheal instillation was performed at 1 mg/kg, and whole protein was extracted from the lungs of the mice over a range of time points (0–24 hr). PGRN expression was analyzed by Western blot analysis, and the results are shown in Figures 1a and 1b. PGRN protein expression started to increase at 6 hr and peaked at 12 hr. Thereafter, the PGRN levels were markedly declined, to reaching their lowest values at 24 hr; however, the levels were still significantly elevated compared to the control. This phenomenon was further confirmed by immunohistochemical staining of the lung tissue, and the PGRN protein was strongly expressed in alveolar macrophages and the alveolar epithelium (Figures 1c and 1d).

### 3.2 Expression of PGRN after ad‐PGRN or ad‐PGRN‐shRNA administration in vivo

**Figure 2:**
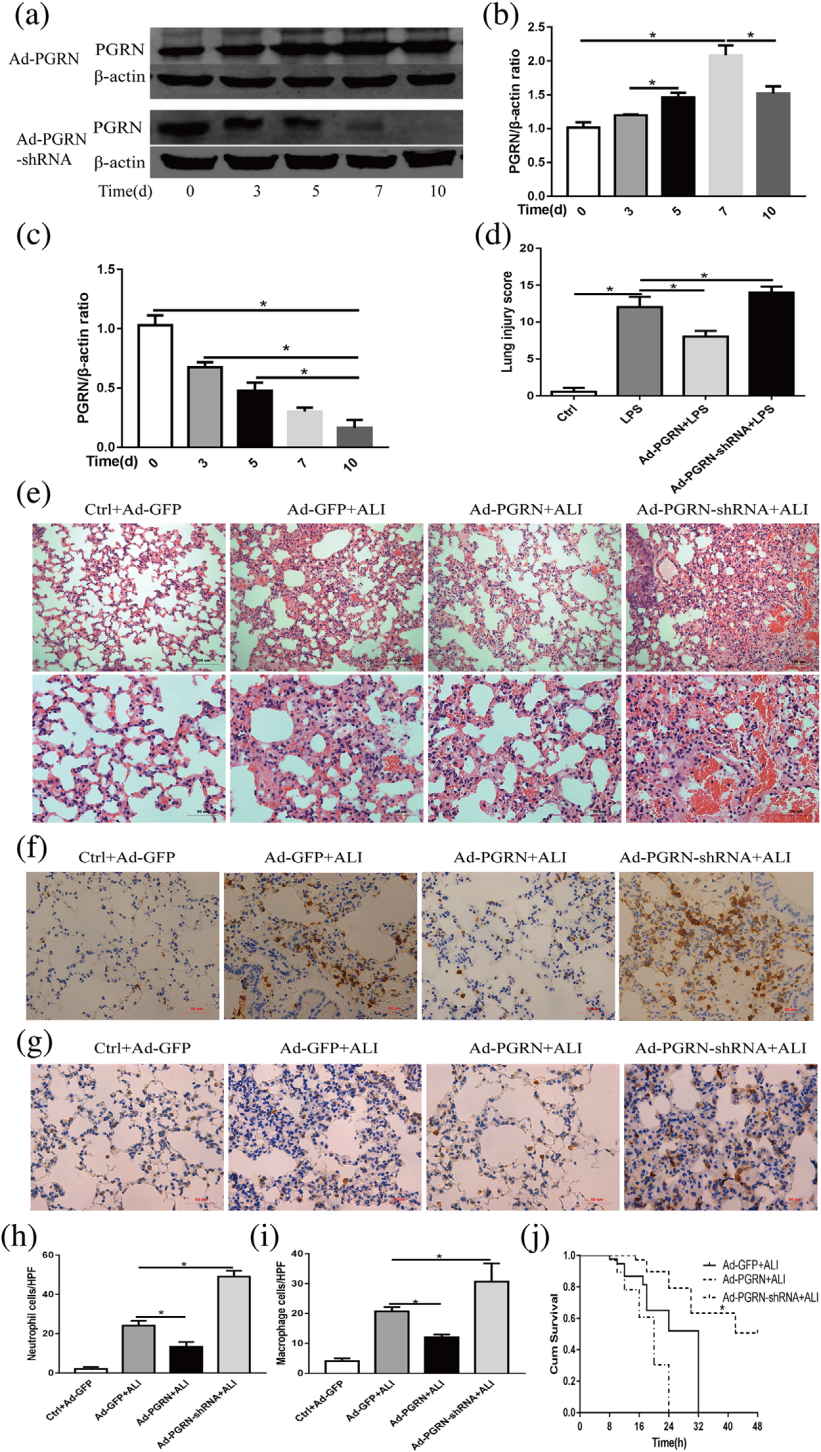
PGRN expression in whole lungs after adenovirus injection in mice. (a) The PGRN protein levels in the mouse lungs at different times after administration of Ad‐PGRN or Ad‐PGRN‐shRNA (1 × 10^9^ PFU) were evaluated by Western blot. (b) Quantitation of the PGRN/β‐actin ratio in the lungs from mice with Ad‐PGRN injection at different times. (c) Quantitation of PGRN/β‐actin ratio in lungs from mice with Ad‐PGRN‐shRNA injection at different times. Effects of PGRN overexpression or knockdown on LPS‐induced lung injury. Mice were subjected to LPS after administration of Ad‐PGRN, Ad‐PGRN‐shRNA, or the NC (Ad‐GFP) for 7 days. (d) Quantitation of the lung injury scores in the different treatment groups. (e) Representative images of lung lesions in the lung sections by HE staining. Scale bar = 100 μm (up), scale bar = 50 μm (down), *n* = 4. (f) Representative images of neutrophil infiltration in lung sections per immunohistochemical staining. Scale bar = 50 μm, *n* = 4. (g) Representative images of macrophage infiltration in lung sections per immunohistochemical staining. Scale bar = 50 μm, *n* = 4. (h) Quantitation of the number of neutrophils per high‐power field (HPF). (i) Quantitation of the number of macrophages per HPF. (j) Mouse survival (*n* = 10) was monitored at different times for 48 hr. *p < 0.05. Data are presented as the mean ± SD

To elucidate the function of PGRN in LPS‐induced lung injury, we first examined whether intratracheal injection of recombinant adenovirus to deliver the PGRN plasmid or shRNA could induce or inhibit PGRN expression in the mouse lung. First, we performed initial experiments with Ad‐PGRN to determine the timing of PGRN expression after intratracheal injection. PGRN protein levels were assessed by Western blot analysis of the lung homogenates 3 days, 5 days, 7 days, and 10 days after Ad‐PGRN administration (1 × 10^9^ PFU). We observed increased PGRN expression levels at 3 days and peak levels at 7 days after Ad‐PGRN administration. Similarly, we examined the inhibitor efficiency of Ad‐PGRN‐shRNA in vivo and found that PGRN protein expression started to decrease at 3 days and decreased maximally at 7 days. Sustained down‐regulation was still observed at 10 days (Figure 2a‐c). In addition, no inflammation or increased mucous production was apparent in our experiment. Therefore, all subsequent studies were performed with 1 × 10^9^ PFU.

### 3.3 Effects of PGRN on LPS‐induced lung injury

After demonstrating the feasibility of inducing or interfering with PGRN expression by airway administration of Ad‐PGRN or Ad‐PGRN‐shRNA, we examined whether enhanced or suppressed PGRN expression could affect the initiation and progression of LPS induced ALI. Histological analysis was performed 24 hr after adenovirus administration. As shown in Figures 2d and 2e, administration of LPS alone into the lungs of the control mice resulted in destruction the lung architecture with inflammation compared to the mice receiving only Ad‐GFP (in which PBS was given intratracheally). However, administration of Ad‐PGRN together with LPS deposition resulted in a significant decrease in the lung injury index compared to the control mice receiving Ad‐GFP together with LPS deposition. Decreases in the lung injury index correlated with decreases in neutrophil and macrophage accumulation in the lungs as determined by immunohistochemical staining (Figure 2f‐i). Histological analysis further showed that mice receiving Ad‐PGRN‐shRNA exhibited enhanced lung injury (Figures 2d and 2e), which was accompanied by increased accumulations of neutrophils and macrophages in the lungs (Figure 2f‐i).

To further evaluate the long‐term effects of intervention in PGRN expression, the mice were subjected to lethal doses of LPS (25 mg/kg) after adenovirus administration, and overall survival was recorded. As shown in Figure 2j, the Ad‐GFP‐treated mice died by 32 hr, but two Ad‐PGRN‐treated mice survived up to 48 hr. The overall survival time was significantly longer in the Ad‐PGRN‐treated group than in the Ad‐GFP group. However, administration of LPS in the Ad‐PGRN‐shRNA groups resulted in opposite effects.

### 3.4 Among the potential miRNAs, miR‐34b‐5p levels are elevated in the lung after LPS administration

To identify miRNAs that potentially targeted PGRN, we first searched the miRNA prediction websites miRanda, TargetScan, and PicTar to screen potential miRNAs targeting PGRN and 12 miRNAs were selected as the best potential candidates. The mechanism by which miRNAs regulate gene expression involves induction of either specific mRNA degradation or translational repression. Therefore, the levels of the candidate miRNAs were measured in the lung homogenates of the mice receiving LPS for 24 hr, which was the time point where the PGRN levels were significantly declined. Three miRNAs were obviously increased (miR‐34b‐5p, 7.3‐fold; miR‐34c‐5p, 3.2‐fold; miR‐34a‐5p, 2.5‐fold), and two miRNAs were significantly decreased (miR‐139‐3p, 0.48‐fold; miR‐466f‐3p, 0.39‐fold), as shown in Table 2. The levels of the remaining miRNAs, namely miR‐4977‐5p, miR‐142b, miR‐449a‐5p, miR‐449b, miR‐764‐3p, miR‐485‐5p, and miR‐7068, showed no significant changes after ALI. The current understanding of miRNAs indicates that miRNAs are negative regulators that modulate targeted mRNA degradation or translational repression. Thus, the up‐regulated miRNAs may play important roles in regulating PGRN. As miR‐34b‐5p of the miR‐34 family is predominantly expressed in the lung (Halappanavar et al., 2011), miR‐34b‐5p may participate in regulating PGRN in ALI.

**Table 2:** The expression of the putative PGRN‐targeting miRNAs in the lung after ALI

miRNA	Fold change (ALI vs. Control)	*p*
miR‐4977‐5p	1.5	0.0987
miR‐142b	1.4	0.0571
miR‐449a‐5p	1.6	0.0546
miR‐34c‐5p	3.2	0.0007
miR‐449b	1.2	0.0535
miR‐764‐3p	1.1	0.2619
miR‐139‐3p	0.48	<0.0001
miR‐34b‐5p	7.3	<0.0001
miR‐485‐5p	1.3	0.1206
miR‐466f‐3p	0.39	<0.0001
miR‐7068	1.4	0.0866
miR‐34a‐5p	2.5	<0.0001

*N* = 6.

### 3.5 MiR‐34b‐5p directly regulates PGRN

**Figure 3:**
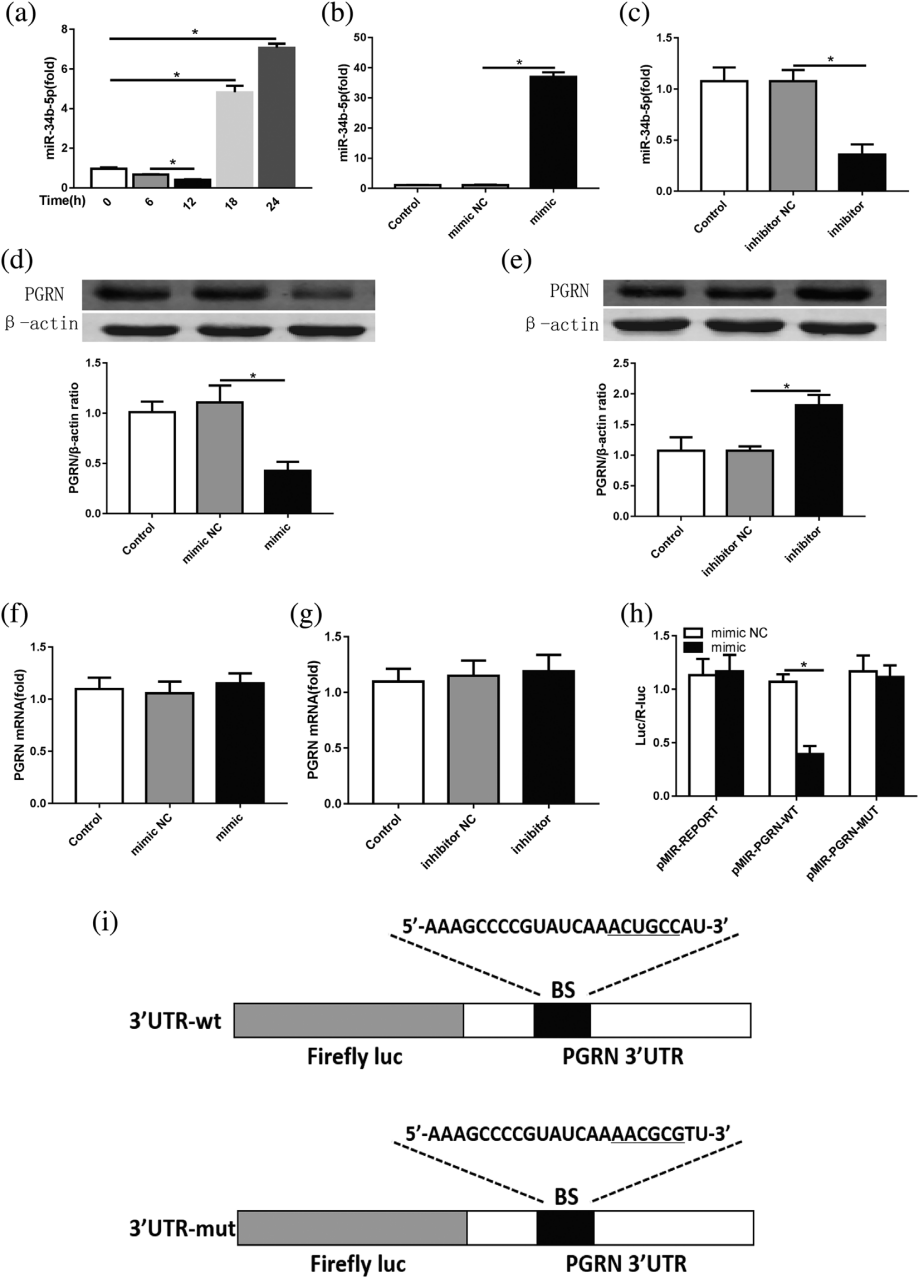
miR‐34b‐5p expression in whole lungs at different times after LPS injection in mice. (a) Quantitation of the miR‐34b‐5p levels in the lungs from mice with LPS injection at different times. The miR‐34b‐5p mimic or inhibitor and corresponding NC were transfected into RAW264.7 cells to validate the relationship between miR‐34b‐5p and PGRN. (b) Quantitation of the transfection efficiency of miR‐34b‐5p mimic. (c) Quantitation of transfection efficiency of miR‐34b‐5p inhibitor. (d,e) The effects of the miR‐34b‐5p mimic and inhibitor on the PGRN protein level in RAW264.7 cells were assessed by Western blot, and β‐actin was as a loading control. (f,g) The effects of the miR‐34b‐5p mimic or inhibitor on PGRN mRNA in RAW264.7 cells were assessed by RT‐PCR, and β‐actin was used as a control. (h) Quantitation of the dual‐luciferase reporter assay, *n* = 6. (i) Schematic of the WT PGRN 3′UTR and mutated PGRN 3′UTR luciferase constructs. *p < 0.05. Data are presented as the mean ± SD

To further evaluate whether the kinetics of the miR‐34b‐5p levels matched those of PGRN expression, the miR‐34b‐5p levels were also assessed in the lung homogenates 0 hr, 6 hr, 12 hr, 18 hr, and 24 hr after LPS administration. Interestingly, miR‐34b‐5p expression was markedly decreased in parallel with PGRN up‐regulation (0–12 hr). Subsequently, miR‐34b‐5p increased in parallel with the PGRN decrease (12 h–24 hr) (Figure 3a).

Because miR‐34b‐5p expression was negatively correlated with PGRN expression in ALI and the PGRN gene had a conservative miR‐34b‐5p seed sequence in its 3′ UTR, we further investigated the correlation between miR‐34b‐5p and PGRN in macrophage. Macrophages are the cell type in which PGRN is predominantly expressed in the lungs, and macrophages have previously been used to imitate the inflammatory response of ALI. The miR‐34b‐5p mimic or inhibitor was transfected into RAW264.7 cells. As a result, the miR‐34b‐5p levels showed significant changes (Figures 3a and 3b). Accordingly, PGRN protein expression was sharply down‐regulated or up‐regulated, but the PGRN mRNA levels were not significantly changed in any intervention group (Figure 3c‐f). These results indicated that miR‐34b‐5p may regulate PGRN expression by suppressing mRNA translation. To further confirm whether miR‐34b‐5p directly targeted PGRN, we co‐transfected the miR‐34b‐5p mimic or NC with luciferase reporters containing WT or mutated miR‐34b‐5p binding sites in the PGRN 3′ UTR into 293T cells. As shown in Figure 3h, luciferase activity was suppressed by miR‐34b‐5p in the WT‐3′ UTR‐containing vectors compared with the NC, whereas mutations in the binding sites of the 3′ UTR‐containing vector abolished the responsiveness to miR‐34b‐5p.

### 3.6 PGRN is a functional target of miR‐34b‐5p

**Figure 4:**
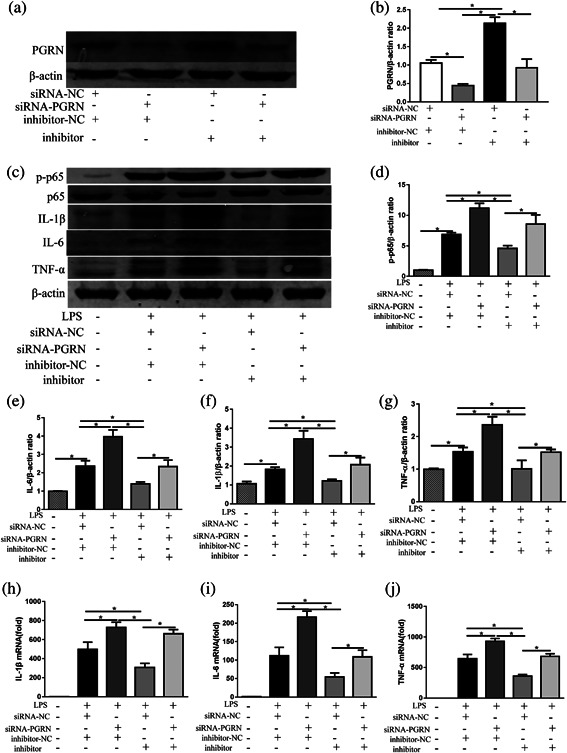
The rescue experiment was performed to confirm the relationship between miR‐34b‐5p and PGRN in vitro. (a) RAW264.7 cells were co‐transfected with the miR‐34b‐5p inhibitor and siRNA‐PGRN, and the protein levels of PGRN were evaluated by Western blot, and β‐actin was used as a loading control. (b) Quantitation of the PGRN/β‐actin ratios in the co‐confected RAW264.7 cells. Inflammatory mediator alterations in RAW264.7 cells after siRNA and/or miR‐34b‐5p inhibitor co‐transfection in LPS conditions (100 ng/ml). (c) The cellular protein levels of phosphorylation of NF‐κB p65, NF‐κB p65, IL‐1β, IL‐6, and TNF‐α were evaluated by Western blot, and β‐actin was used as a loading control. Quantitation of (d) the p‐p65/β‐actin ratios, (e) IL‐6/β‐actin ratios, (f) IL‐1β/β‐actin, and (g) TNF‐α/β‐actin ratios. The cellular levels of (h) IL‐1β mRNA, (i) il‐6 mRNA, and (j) TNF‐α mRNA in cells were determined by RT‐PCR. *p < 0.05. Data are presented as the mean ± SD

To verify that miR‐34b‐5p enhanced macrophages sensitivity to LPS by negatively regulating PGRN expression, we performed a “rescue” experiment to investigate the effect of miR‐34b‐5p in the presence of PGRN inhibition in activated RAW264.7 cells. As shown in Figures 4a and 4b, the results of the rescue experiment showed that co‐transfection with PGRN siRNA markedly abolished the ability of miR‐34b‐5p inhibitor to enhance PGRN expression in RAW264.7 cells. LPS‐induced increase in protein levels of IL‐1β, IL‐6, and TNF‐α was dramatically enhanced by co‐treated with LPS and PGRN siRNA, while the ability of miR‐34b‐5p inhibitor to suppress these inflammatory mediators expression was abolished by co‐transfection of PGRN siRNA and miR‐34b‐5p inhibitor (Figures 4c and 4e‐g). Similar results were also observed at the mRNA levels (Figure 4h‐j). In addition, the protein level of the phosphorylation of NF‐κB P65 subunit was significantly alleviated by co‐confection with miR‐34b‐5p inhibitor, while co‐transfection of PGRN siRNA could neutralize the effects in LPS conditions (Figures 4c and 4d).

### 3.7 Effects of miR‐34b‐5p inhibition on LPS‐induced lung injury in vivo

**Figure 5:**
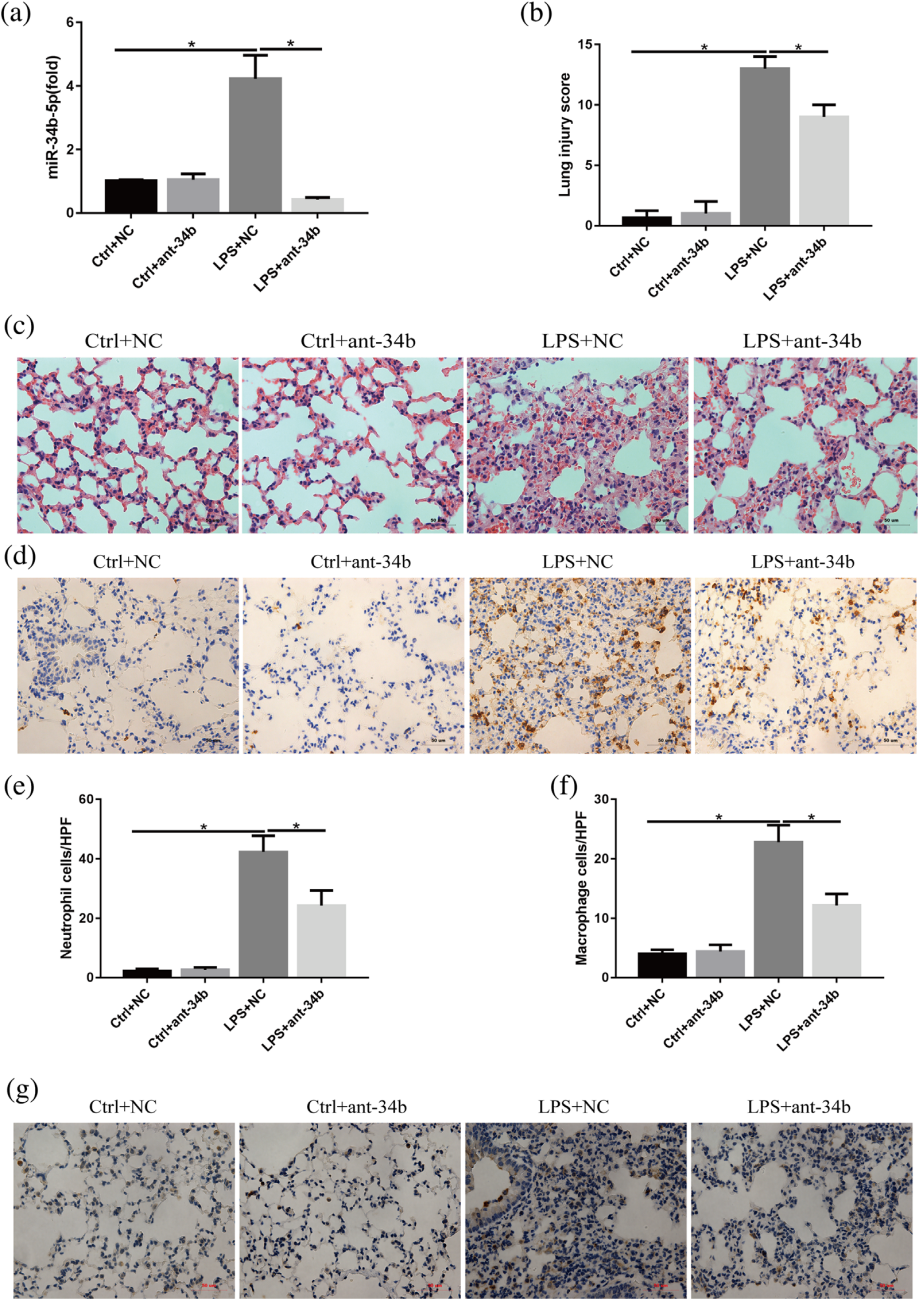
Effects of miR‐34b‐5p inhibition on LPS‐induced lung injury. Mice were subjected to LPS after administration of the miR‐34b‐5p antagomir or NC for 48 hr. (a) Quantitation of the transfection efficiency of the miR‐34b‐5p antagomir in vivo. (b) Quantitation of the lung injury scores in the different treatment groups. (c) Representative images of lung lesions in the lung sections per HE staining. Scale bar = 50 μm, *n* = 4. (d) Representative images of neutrophils infiltration in lung sections by immunohistochemical staining. scale bar = 50µm, *n* = 4. (e) Quantitation of the number of neutrophils per HPF. (f) Quantitation of the number of macrophages per HPF. (g) Representative images of macrophage infiltration in lung sections per immunohistochemical staining. Scale bar = 50 μm, *n* = 4. *p < 0.05. Data are presented as the mean ± SD

**Figure 6:**
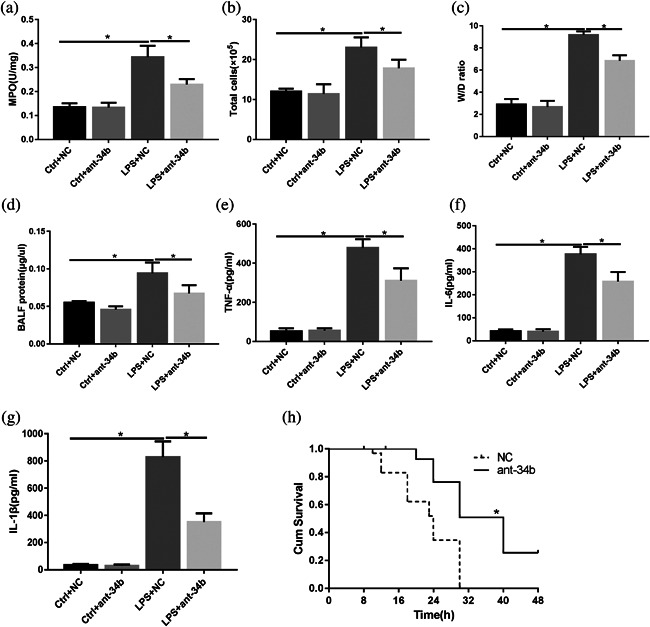
miR‐34b‐5p inhibition significantly decreases inflammatory mediator levels in the BALF and improves the survival ratio during ALI. Mice were subjected to LPS after administration of the miR‐34b‐5p antagomir or NC for 48 hr. (a) MPO in the lungs was assessed by ELISA. (b) The total number of BALF cells and neutrophils was determined with cytospin and a hemocytometer. (c) Lung W/D ratios. (d) The protein concentrations in the BALF were determined with BCA assays. The levels of (e) TNF‐α, (f) IL‐6, (g) IL‐1βin the BALF. (h) Mouse survival (*n* = 10) was monitored at different times for 48 hr. *p < 0.05. Data are presented as the mean ± SD

To further clarify the function of miR‐34b‐5p in ALI, the miR‐34b‐5p antagomir was injected into mice through the tail vein to generate miR‐34b‐5p‐silences mice. As shown in the Figure 5a, the mice receiving the miR‐34b‐5p antagomir exhibited lower miR‐34b‐5p levels by RT‐PCR. Administration of the miR‐34b‐5p antagomir alone into the control mice (in which PBS was administered intratracheally) did not result in a significant change in the lung injury index compared with mice receiving only the antagomir NC. However, administration of the miR‐34b‐5p antagomir together with LPS deposition caused a significant decrease in the lung injury index compared with the mice receiving the antagomir NC together with LPS deposition (Figures 5b and 5c). As a result, the decreases in the lung injury index were accompanied by decreases in the accumulation of neutrophils and macrophages in the lungs as determined by immunohistochemical staining (Figure 5d‐g). This phenomenon was further confirmed with the MPO assay and total cell analysis of the BALF (Figures 6a and 6b).

We further examined the W/D ratio to determine whether mice receiving the miR‐34b‐5p antagomir exhibited lower vascular permeability index than in the ALI group. As shown in Figure 6c, the mice receiving the miR‐34b‐5p antagomir showed a marked decrease in the permeability index compared with the ALI group. Regarding albumin leakage, the protein content of the BALF was significantly higher in the mice receiving LPS than in the mice receiving the miR‐34b‐5p antagomir during lung injury (Figure 6d). We also examined the effects of miR‐34b‐5p inhibition on the levels of pro‐inflammatory mediators in the BALF after LPS deposition. As shown in Figure 6e‐g, the levels of IL‐6, TNF‐α, and IL‐1β in the BALF from the lungs of the mice receiving the miR‐34b‐5p antagomir after administration of LPS were significantly decreased compared with the BALF from the lungs treated with the antagomir NC plus LPS.

To assess the sensitivity of the miR‐34b‐5p‐deficient mice to LPS‐induced lung injury, the mice that were pretreated with the miR‐34b‐5p antagomir or antagomir NC were subjected to lethal doses of LPS (25 mg/kg), and overall survival was recorded (Xu, Zhu, et al., 2015). As shown in Figure 6h, the mortality rates after the LPS treatment were 100% for the WT mice and 80% for the miR‐34b‐5p^−/−^ mice.

### 3.8 Effects of miR‐34b‐5p inhibition on lung cell apoptosis in mice after LPS administration

**Figure 7:**
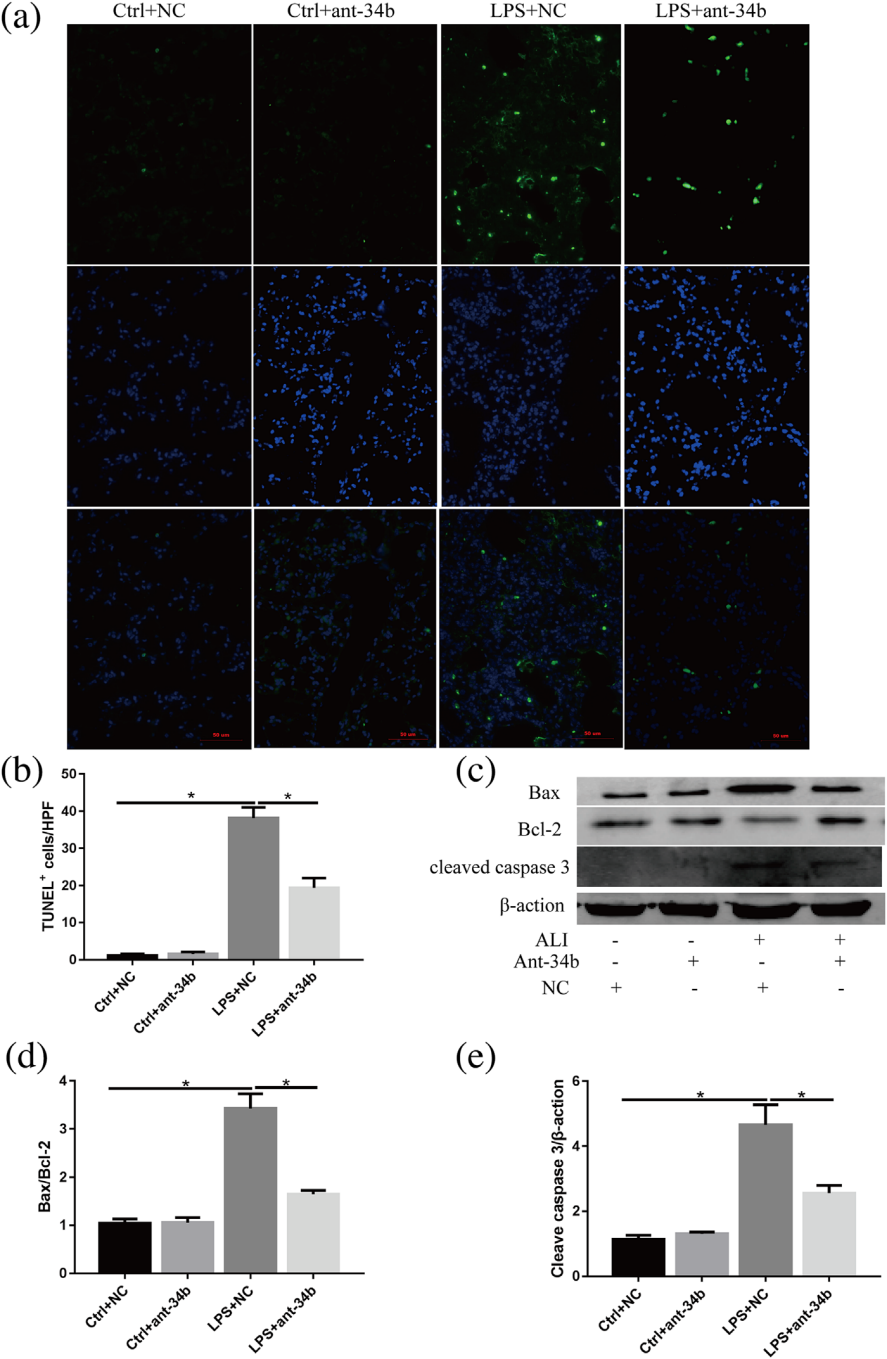
Effect of miR‐34b‐5p inhibition on lung cell apoptosis and apoptosis‐associated protein expression in LPS‐induced ALI mouse model. (a) Representative images of lung apoptosis in lung sections by TUNEL assay. Nuclei were observed by DAPI staining. Scale bar = 50 µm, *n* = 4. (b) Quantitation of the number of TUNEL‐positive (dead) cells (number/HPF) in the different groups. (c) The protein levels of Bax, Bcl‐2, and cleaved‐caspase‐3 were evaluated by Western blot, and β‐actin was used as a loading control. (d,e) Quantitation of the Bax/Bcl‐2, cleaved‐caspase‐3/β‐actin ratios in the lungs from the mice with LPS and/or the miR‐34b‐5p antagomir. *p < 0.05. Data are presented as the mean ± SD

To determine whether miR‐34b‐5p inhibition affected lung cell apoptosis during ALI, we evaluated lung apoptosis with TUNEL assays. TUNEL‐positive cells were increased in the lungs of the mice after LPS administration, but the number of TUNEL‐positive cells was significantly decreased in the lungs of the mice receiving the miR‐34b‐5p antagomir after LPS administration (Figures 7a and 7b). We further measured the levels of important proteins involved in apoptotic pathways. The levels of Bax and cleaved‐caspase‐3 were greater in the lungs of the mice with the antagomir NC than in the lungs with the miR‐34b‐5p antagomir after LPS administration, but the Bcl‐2 protein levels were significantly increased in the mice receiving the miR‐34b‐5p antagomir together with LPS compared with the mice receiving LPS and the NC (Figure 7c‐e).

### 3.9 Protective effects of miR‐34b‐5p inhibition depend on PGRN in vivo

**Figure 8:**
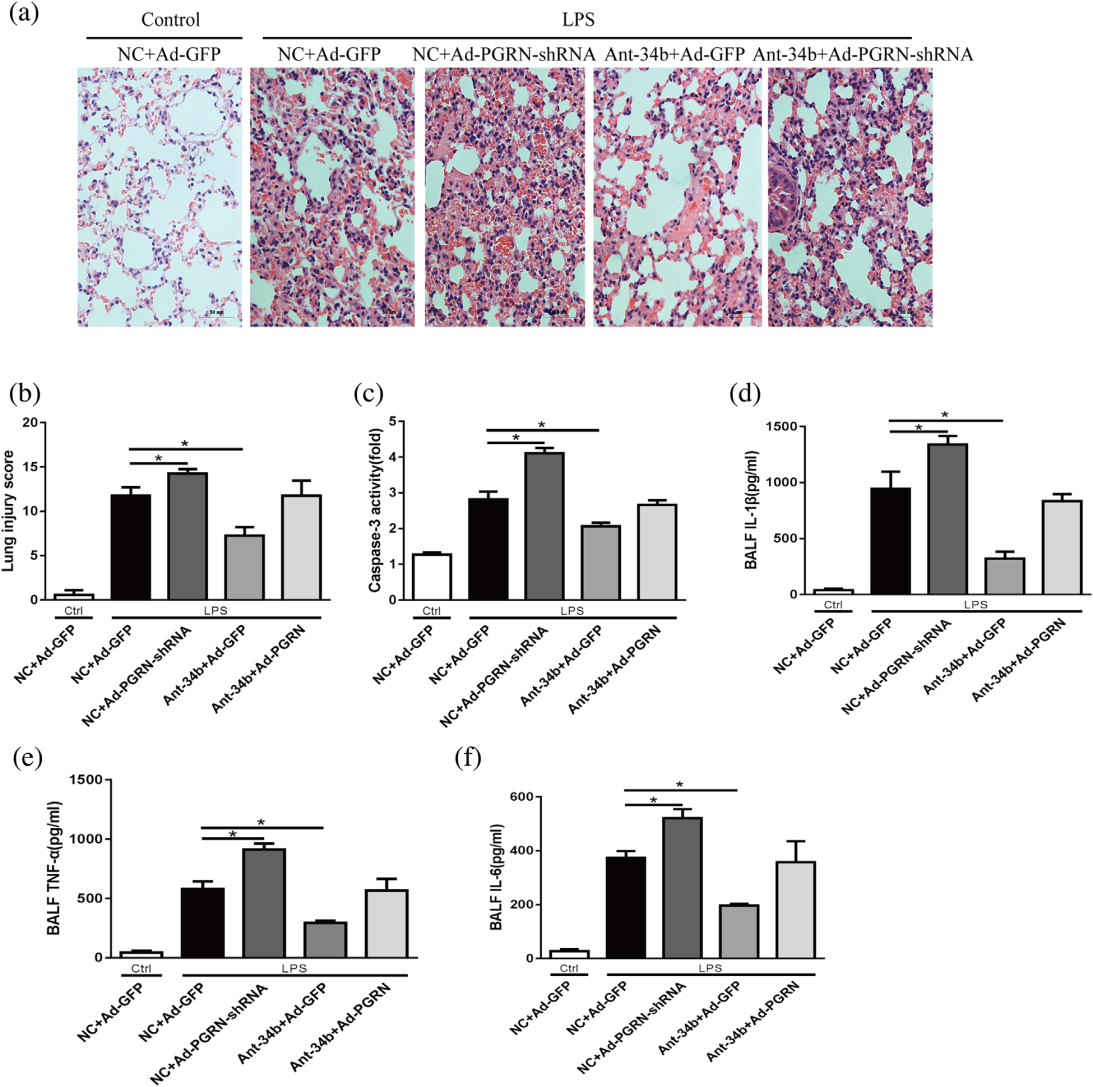
The protective effects of miR‐34b‐5p are dependent on PGRN. Mice were subjected to the miR‐34b‐5p antagomir after administration of Ad‐PGRN‐shRNA or the NC (Ad‐GFP) for 7 days. After 48 hr, the mice were administered with LPS by intratracheal injection for 24 hr. (a) Representative images of the lung lesions in the lung sections by HE staining. Scale bar = 50 µm, *n* = 4. (b) Quantitation of the lung injury scores in the different treatment groups. (c) The caspase‐3 activity of the lungs in the different treatment groups, *n* = 4. The levels of (d)IL‐1β, (e)TNF‐α, and (f) IL‐6 in the BALF were assessed by ELISA. *p < 0.05. Data are presented as the mean ± SD

Nest we ascertained whether the special relationship between miR‐34b‐5p and PGRN still existed in vivo. After co‐confecting Ad‐PGRN‐shRNA and the miR‐34b‐5p antagomir in vivo, the miR‐34b‐5p antagomir displayed similarly protective effects in the groups without PGRN inhibition, further confirming that miR‐34b‐5p inhibition was useful for ALI. Interestingly, the miR‐34b‐5p antagomir did not prevent ALI‐induced lung lesions in the mice, inflammatory mediator production or apoptosis activation when the mice were subjected to Ad‐PGRN‐shRNA (Figure 8). Taken together, these data indicate that PGRN is necessary for miR‐34b‐5p inhibition‐mediated protective effects in ALI.

## 4 DISCUSSION

ALI is associated with high morbidity and mortality rates worldwide and remains a major challenge for clinicians. Increasing studies have demonstrated that PGRN plays important roles in apoptosis and inflammation. However, the biological function of PGRN in ALI and the identities of the miRNAs that regulate PGRN remain unclear. In this study, we provide the first evidence that PGRN expression is dynamically changed during ALI and these changes play a critical role in the inflammatory response and apoptosis in the lungs. Among the miRNAs targeting PGRN, miR‐34b‐5p was dramatically up‐regulated in the lung homogenates and could directly regulate PGRN. Furthermore, miR‐34b‐5p knockdown exerted protective effects for attenuating inflammatory mediator production, inflammatory cells infiltration, epithelial cell apoptosis, the lung lesion, and improving survival. The protective effects of miR‐34b‐5p inhibition require for PGRN during ALI in vitro and in vivo. This is the first to investigate the roles of miR‐34b‐5p in regulating the critical pathogenic steps, namely, the inflammatory response, and apoptosis, in ALI.

PGRN is a potent anti‐inflammatory growth factor. Elevated PGRN levels are tightly relevant to multiple inflammatory diseases, such as arthritis (Fu et al., 2017), type two diabetes (Xu, Zhou, et al., 2015), osteonecrosis (Han et al., 2017), in which PGRN inhibits inflammation and is associated with poor prognosis. Interestingly, a reduction in PGRN expression has been observed in an acute kidney injury mouse model after ischemia/reperfusion and in the cerebrospinal fluid of patients with subarachnoid hemorrhage (Zhou, Tang, et al., 2015; Zhou, Xie, et al., 2015), suggesting that PGRN expression in inflammatory disorders may be, in part, dependent on the particular disease model. In pulmonary inflammatory diseases, PGRN overexpression is also apparent in the lung tissues of a mouse endotoxic shock model (Yu et al., 2016), in the sera of patients with sepsis (Song et al., 2016), and in the BALF from patients with community‐acquired pneumonia (Zou et al., 2017). However, the levels of PGRN in the BALF are decreased in mice on day three after induction with of ALI by intraperitoneal injection of LPS (Guo et al., 2012). In contrast to these studies, we measured a progressive up‐regulation of PGRN during the early phase of ALI, and the PGRN levels progressively declined, reaching a minimum level at 24 hr after LPS administration; however, the level was still elevated compared with the control groups, which may be due to the body's response to ALI. This phenomenon implies that there is a negative feedback mechanism that regulates PGRN expression during ALI.

Next, we performed gain‐ and loss‐of‐function experiments by intratracheal injection of recombinant adenovirus to deliver the PGRN plasmid or shRNA into the lungs to investigate the effects of PGRN on ALI. Previous studies have shown that adenovirus is an ideal candidate vector for gene delivery because of its exclusive advantages; adenovirus infections are efficient, adenovirus has a high carrying capacity, and adenovirus does not integrate into the host genome (Chen, Gao, et al., 2016; Reynolds, Holmes, Danilov, & Reynolds, 2012). Neutrophils are recruited to the alveolar space and lung interstitium, thereby producing large quantities of chemokines, such as chemokines KC (CXCL‐1) and MIP‐2 (CXCL‐2) (Lerner, Lei, Sundar, & Rahman, 2016; Rancan et al., 2017), and reactive oxygen species (ROS) (Spassov et al., 2017), and neutrophils play critical roles in the development of ALI. Alveolar macrophages also play important roles in the release many pro‐inflammatory cytokines, such as IL‐1β, TNF‐α, and IL‐6 (Parpaleix et al., 2016; Yan et al., 2014). In this study, the functional analysis demonstrated that both Ad‐PGRN and Ad‐PGRN‐shRNA caused significant changes in PGRN expression in vivo and that these changes were sustained for a long time. In addition, the adenoviral vectors did not cause obvious lung inflammation at a dose of 1 × 10^9^ PFU. Furthermore, the gain‐ and loss‐of‐function experiments demonstrated that PGRN could improve survival and relieve lung inflammation, namely, accumulation of neutrophils, and macrophages into the lungs. In addition, accumulative studies have indicated that PGRN deficiency is also closely associated with macrophage infiltration and activation (Lui et al., 2016). Unfortunately, the knowledge about PGRN downstream remains unclear. Some studies have reported that the biological effects of PGRN require for Sortilin, TNFR1, TNFR2, and EphA2 (Hu et al., 2010; Krabbe et al., 2017; Neill et al., 2016). In fact, the anti‐inflammatory effects of PGRN might be mediated, at least in part, by blocking TNF‐α production via binding to TNFR receptors. In our previous study, we also found that the PGRN/TNFR2 interaction was crucial for the protective effect of PGRN on LPS‐induced ALI (Guo et al., 2012). Thus, the regulation of PGRN may serve as a target for the treatment of ALI.

miRNAs constitute a class of highly conserved, noncoding RNAs that contain 19–22 nucleotides, are distributed widely in animals, plants, and microorganisms, and participate in different pathophysiological processes (Cui, Zhou, Ross, & Zempleni, 2017). Over the past decade, accumulative data have demonstrated that aberrant expression of miRNAs involved in the occurrence and development of ALI (Cai et al., 2012; Fang, Gao, Hao, & Liu, 2017). During ALI, the expression levels of miR‐7, miR‐582‐5p, miR‐582‐3p, miR‐450a‐3p, and miR‐29b are up‐regulated (Guo et al., 2013; Zhao et al., 2016), but the expression levels of miR‐125b, miR‐208a, miR‐133b, let‐7, miR‐34c are down‐regulated (Guo et al., 2013; Otsuki, Ishikawa, Hori, Goto, & Sakamoto, 2015). PGRN can be regulated by miRNAs in different physiological and pathological contexts (Jiao, Herl, Farese, & Gao, 2010; Qian et al., 2016). Previous studies have shown that miR‐107 and miR‐659 inhibit PGRN in response to traumatic brain injury and frontotemporal dementia, respectively (Rademakers et al., 2008; Wang et al., 2010). In addition, miR‐29b‐3p directly regulates PGRN, promotes chondrocyte apoptosis, and facilitates the occurrence and development of osteoarthritis (Chen et al., 2017). In the present study, the bioinformatics analysis, dual‐luciferase reporter assays, rescue experiments, and Western blot analysis demonstrated that PGRN was a direct downstream target of miR‐34b‐5p and that an inverse relationship existed between miR‐34b‐5p and PGRN. To the best of our knowledge, this study is the first to demonstrate that PGRN is the functional target gene of miR‐34b‐5p. Interestingly, we also found the expression levels of miR‐34b‐5p and PGRN was both up‐regulated in the lungs from the mice with ALI at 24 hr, and we hypothesized that PGRN was not only regulated by miR‐34b‐5p but also modulated by various factors, such as TMEM106B and IL‐6 (Finch et al., 2011; Liu, Zhang, et al., 2016), indicating that other mechanisms may involve in the regulation of PGRN expression during ALI. We believe that elevated PGRN levels in the lungs are a comprehensive result of multiple factors, and the final seemingly positive correlation may mask its complicated mechanism.

The miR‐34 family includes miR‐34a, miR‐34b, and miR‐34c, which differ from each another by only two or three bases, and these miRNAs play diverse roles that are target dependent (He et al., 2007). Up‐regulation of miR‐34a‐5p expression suppresses SIRT1 and regulates epithelial apoptosis and ROS production in ischemia/reperfusion injury (Wang, Yao, et al., 2016). The miR‐34 family negatively regulates HMGB1, Wnt, and Notch signaling (Bettinsoli et al., 2017; Kim et al., 2011; Liu, Ren, & Chen, 2017). A previous study demonstrated that the expression of miR‐34a, but not miR‐34b, was significantly decreased in spinal cord injury and that miR‐34a performed critical functions in spinal cord injury by targeting Notch1 (Chen, Cao, et al., 2016), indicating that miR‐34 family may play an important role in acute inflammation. Here, we have described the effects of miR‐34b‐5p in the development of ALI. In vitro, the results of the rescue experiments showed that miR‐34b‐5p inhibition ameliorated the LPS‐induced inflammatory response. In vivo, miR‐34b‐5p silencing with an antagomir, which is nucleic acid analog that shows high specificity for its target RNA, leading to high efficient gene silencing (Wang et al., 2012), can protect mice from ALI by attenuating inflammatory mediator generation, inflammatory cell infiltration, and epithelial cell apoptosis, further improving lung lesions and survival. Although the levels of miR‐34b‐5p displayed at low expression in this study, the functional experiment showed that miR‐34b‐5p inhibition provides protective effects in vivo and in vitro, indicating that miR‐34b‐5p may play biological effects in the development of ALI. In addition, we used the methods that generate ALI model by intratracheal instillation of LPS instead of cecal puncture also affects the abundance of miR‐34b‐5p. Therefore, this study is the first to demonstrate the role of miR‐34b‐5p in inflammatory diseases, particularly ALI.

Notably, excessive apoptosis of alveolar epithelial cells is a main factor in disease progression in ALI (Hu et al., 2014). Increased production and release of inflammatory mediators, such as TNF‐α, IL‐6, IL‐1β, and ROS, ultimately induce cell death by apoptosis and pyroptosis (Lv et al., 2017; Zhang et al., 2016). Thus, the suppression of epithelial apoptosis may serve provide targets for the treatment of ALI. The miR‐34b‐5p family may have functions in alveolar epithelial apoptosis. miR‐34b‐5p is significantly down‐regulated and involved in cell apoptosis in non‐small‐cell lung cancer via targeted inhibition of Met (Wang et al., 2013). In addition, down‐regulation of miR‐34b influences epithelial cell apoptosis associated with conditions involving cigarette smoke (Izzotti et al., 2009). These studies show that miR‐34b‐5p may also have functions in alveolar epithelial cell apoptosis. However, in this study, we demonstrated that the levels of miR‐34b‐5p were progressively up‐regulated during ALI and that the miR‐34b‐5p knockdown alleviated caspase‐3 activation and suppressed apoptosis, implying that the protective effects of miR‐34b‐5p inhibition may be associated with relieving LPS‐induced PGRN suppression. This result is consistent with the elevated miR‐34b‐5p levels observed in the hippocampal astrocyte tissue of a rat recurrent seizure model accompanied by accumulative astrocyte apoptosis (Liu, Liu, et al., 2016). Thus, the effect of miR‐34b‐5p in response to apoptosis may depend on the cellular context.

In summary, our study has shown that miR‐34b‐5p inhibition attenuates lung inflammation and apoptosis in an LPS‐induced ALI mouse model by targeting progranulin. We have characterized the role of the miR‐34b‐5p/PGRN axis in the pathogenesis of ALI and confirmed that miR‐34b‐5p is a novel miRNA that regulates PGRN in the lung. This study has shown that miR‐34b‐5p and PGRN may be potential targets for ALI treatments.

## CONFLICTS OF INTEREST

The authors declare that there are no conflict of interest regarding the publication of this paper.
